# A Single L/D-Substitution at Q4 of the mInsA_2-10_ Epitope Prevents Type 1 Diabetes in Humanized NOD Mice

**DOI:** 10.3389/fimmu.2021.713276

**Published:** 2021-08-30

**Authors:** Mengjun Zhang, Yuanqiang Wang, Xiangqian Li, Gang Meng, Xiaoling Chen, Lina Wang, Zhihua Lin, Li Wang

**Affiliations:** ^1^Department of Pharmaceutical Analysis, College of Pharmacy, Army Medical University (Third Military Medical University), Chongqing, China; ^2^Institute of Immunology People’s Libration Army (PLA) & Department of Immunology, Army Medical University (Third Military Medical University), Chongqing, China; ^3^Department of Pharmaceutical Engineering, School of Pharmacy and Bioengineering, Chongqing University of Technology, Chongqing, China; ^4^Department of Pathology, Southwest Hospital, Army Medical University (Third Military Medical University), Chongqing, China; ^5^Department of Immunology, Weifang Medical University, Weifang, China

**Keywords:** type 1 diabetes, altered peptide ligand, D-amino acid substitution, mInsA_2-10_, NOD.*β2m^null^.HHD* mice

## Abstract

Autoreactive CD8^+^ T cells play an indispensable key role in the destruction of pancreatic islet β-cells and the initiation of type 1 diabetes (T1D). Insulin is an essential β-cell autoantigen in T1D. An HLA-A*0201-restricted epitope of insulin A chain (mInsA_2-10_) is an immunodominant ligand for autoreactive CD8^+^ T cells in NOD.*β2m^null^*.*HHD* mice. Altered peptide ligands (APLs) carrying amino acid substitutions at T cell receptor (TCR) contact positions within an epitope are potential to modulate autoimmune responses *via* triggering altered TCR signaling. Here, we used a molecular simulation strategy to guide the generation of APL candidates by substitution of L-amino acids with D-amino acids at potential TCR contact residues (positions 4 and 6) of mInsA_2-10_, named mInsA_2-10_DQ4 and mInsA_2-10_DC6, respectively. We found that administration of mInsA_2-10_DQ4, but not DC6, significantly suppressed the development of T1D in NOD.*β2m^null^*.*HHD* mice. Mechanistically, treatment with mInsA_2-10_DQ4 not only notably eliminated mInsA_2-10_ autoreactive CD8^+^ T cell responses but also prevented the infiltration of CD4^+^ T and CD8^+^ T cells, as well as the inflammatory responses in the pancreas of NOD.*β2m^null^.HHD* mice. This study provides a new strategy for the development of APL vaccines for T1D prevention.

## Introduction

Type 1 diabetes (T1D) is a spontaneous organ-specific autoimmune disease characterized by T cell-mediated elimination of insulin-producing pancreatic islet β-cells. Given the close association between major histocompatibility complex (MHC) class II molecules and type 1 diabetes has been early found (1), β-cell autoreactive CD4^+^ T cells were the most intensively studied in both humans and NOD mice (2). However, the importance of β-cell autoantigen-specific CD8^+^ T cells in the pathogenesis of type 1 diabetes has been heightened by multiple studies in NOD mice (3–5). Either CD8α or β2-microglobulin-deficient NOD mice do not develop diabetes (6). Similarly, diabetes does not occur in NOD mice depleted of CD8^+^ T cells by antibody treatment (7). Human leukocyte antigen-A*0201 (HLA-A*0201), one of the most commonly expressed MHC class I allele in Caucasians and Asians (50%), has been also indicated to contribute to the susceptibility to T1D (8). NOD.*β2m^null^*.*HHD* mice, carrying human HLA-A*0201 but no murine MHC class I molecules, show significantly accelerated T1D onset (8). Thus, induction of β-cell autoreactive CD8^+^ T-cell tolerance has been considered as a promising approach for the prevention of T1D (9).

Insulin is a primary β-cell autoantigen that initiates spontaneous T1D in both NOD mice and human (10), so induction of insulin-autoreactive T-cell tolerance can lead to prevention of T1D (11). Multiple CD8^+^ T cell epitopes of insulin such as A chain 2-10 (mInsA_2-10_) and B chain 5-14 (mInsB_5-14_), which are identified to be HLA-A*0201-restricted and immunodominant in NOD.*β2m^null^*.*HHD* mice with high potential T1D relevance, represent important candidates of CD8^+^ T cell targets in human T1D patients.

Altered peptide ligands (APLs) carrying amino acid substitutions at T cell receptor (TCR) or MHC contact positions can trigger altered TCR signaling events and are suggested as useful tools to modulate autoimmune responses. For examples, administration of APLs of a known immunodominant CD8^+^ T cell epitope with partial agonist activity (12) or nanoparticles coated with APL-MHCs complexes (13) has been shown to effectively induce autoreactive CD8^+^ T cell tolerance and prevent T1D in NOD mice. We recently found that repeated treatment of APL of mInsB_5–14_ with histidine to phenylalanine substitution at the potential TCR contact site (p6) prevents T1D *via* selectively expanding a tiny population of CD8^+^CD25^+^Foxp3^+^ regulatory T cells in humanized NOD mice (14).

The introduction of D-amino acids into the sequence of peptides is widely used to improve the stability and prevent peptides from proteolytic degradation (15). On the other hand, scanning with D-amino acid has been previously exploited for molecular analysis of MHC binding or/and TCR interacting residues within a T cell epitope, since the corresponding single D-amino acid substitution minimizes the influence of charges of each original residue as well as the size and molecular weight of the native peptide (16–18). Although these limited earlier studies have shown that T cell priming capacity of a T cell epitope can be altered by introducing D-amino acid at selected residues, whether APLs derived from D-amino acid substitution in TCR contact residues of native peptide are suitable for prevention of autoimmune diseases has been rarely considered. Here, we designed two APL candidates by *in silico*-assisted substitution of L-amino acids with D-amino acids at positions 4 and 6 of mInsA_2-10_, respectively, which are in close contact with the TCR and potentially important for the recognition and response of specific T cells. We found that these two APLs, mInsA_2-10_DQ4 and DC6, significantly inhibited the native mInsA_2-10_ peptide-induced proliferation of splenocytes from NOD.*β2m^null^.HHD* mice *in vitro*. However, *in vivo* administration of mInsA_2-10_DQ4 but not DC6 notably reduced the insulitis and effectively delayed the development of T1D in NOD.*β2m^null^.HHD* mice. Mechanistically, systemic treatment with mInsA_2-10_DQ4 blinded autoreactive CD8^+^ T cell responses toward to mInsA_2-10_ and reduced the infiltration of both autoreactive CD4^+^ T and CD8^+^ T cells into the pancreas in NOD.*β2m^null^.HHD* mice.

## Materials and Methods

### Mice

NOD.*β*2*m^null^.HHD* mice were purchased from the Jackson Laboratory (Bar Harbor, ME, USA). All mice were bred and maintained in specific pathogen-free facilities and handled according to “Principles of Laboratory Animal Care and Use in Research” (Ministry of Health, Beijing, China). All experimental protocols were approved by the Animal Ethics Committee of the Army Medical University (Third Military Medical University).

### Blood Glucose Monitoring

Blood glucose was monitored using a glucometer (OneTouch Ultral; LifeScan, Milpitas, CA, USA) at weekly intervals, beginning at 10 weeks of age. Diabetes was defined as two consecutive blood glucose values above 11.1 mM.

### Epitope Modification and Molecular Dynamics Simulation

We constructed the HLA-A*0201/mInsA_2-10_(IVDQCCTSI) complex based on the crystal structure (PDB ID: 3MRE) by mutating of epitope, which was used as template to build other complexes by replacing the L-amino acid of mInsA_2-10_ with the corresponding D-amino acid at the assigned position (listed in **Table 1**). Subsequently, the epitopes complexed to HLA-A*0201 were put into a cubic box with 0.15 M NaCl solution and kept the ensemble neutral, then 120 ns (100 ns for HLA-A*0201/epitope) molecular dynamics simulation was performed with ff14SB force filed by AMBER 16 (19, 20). The root means square deviation (RMSD) of backbone (C_α_, C, N, O of main chain) between sampling and initial conformation of HLA-A*0201 and epitopes was used to monitor the states of MD simulation. The ensemble reached the stable state while the RMSD was fluctuating at any number for a long time (more than 20 ns), then the MD simulation was ended. The binding free energy between epitopes (included mInsA_2-10_) and HLA-A*0201 was calculated with molecular mechanics/Poisson Boltzmann surface area (MM/PBSA) method for the stable conformations extracted from last 20 ns trajectory, which were used to evaluate their potential affinity (21). Sequentially, we averaged the number of hydrogen bonds between epitopes and HLA-A*0201 and the solvent accessible surface area (SASA) of epitope with the LCPO model for stable conformations (22).

**Table 1:** The distance (P1–P9), average hydrogen bonds, and binding energy of epitopes.

Epitope	Sequence	Distance (Å, P1-P9)	HBonds (average)	Binding Energy (kcal/mol)
				MHC/Epitope
mInsA_2-10_	IVDQCCTSI	23.52	12	−4986.95
mInsA_2-10_DQ4	IVD(D-Gln)CCTSI	23.43	6	−4974.66
mInsA_2-10_DC6	IVDQC(D-Cys)TSI	19.81	6	−4616.46

### Peptides and Mice Treatment

Synthetic peptides mInsA_2-10_ (IVDQCCTSI), OVA_257-264_ (SIINFEKL), HIV pol_476-484_ (ILKEPVHGV), mInsA_2-10_DC6 (IVDQC(D-Cys)TSI), and mInsA_2-10_DQ4 (IVD(D-Gln)CCTSI) were synthesized with purity >95% at Chinese Peptide Company (Hangzhou, China). Cohorts of 4-week-old female NOD.*β*2*m^null^.HHD* mice were intraperitoneally injected with 100 μg (1 μg/μl) in PBS, and this procedure was repeated every week until the sixth injection.

### Histology

Pancreatic tissues from 12-week-old non-diabetic female NOD.*β*2*m^null^.HHD* mice immunized with different peptides (10 mice per group) were fixed in 10% neutral-buffered formalin, and the paraffin-embedded samples were stained with hematoxylin and eosin (H&E). A minimum of 10 islets from each mouse were microscopically observed by two different observers, and insulitis scoring was performed according to the following criteria: 0, no infiltration; 1, peri-insulitis; 2, insulitis with <50% islet area infiltrated; 3, insulitis with >50% islet area infiltrated.

### HLA-A*0201 Binding Assay

T2 cells (1×10^6^ cells/ml) were incubated with each peptide (50 µg/ml) in serum-free RPMI 1640 medium supplemented with 3 µg/ml β-2-microglobulin (Sigma-Aldrich, St. Louis, MO, USA) for 16 h at 37°C. Then cells were washed and stained with anti-HLA-A2 mAb BB7.2 (purified in-house from the hybridoma obtained from ATCC), followed by incubation with FITC-conjugated goat anti-mouse IgG (Beyotime, Jiangsu, China), and analyzed using an FACSAria™ instrument (BD Bioscience, Franklin Lakes, NJ, USA).

### Mouse IFN-γ ELISPOT Assays

ELISPOT plates were precoated with anti-mouse mAb (MabTech, Stockholm, Sweden) overnight at 4°C, and blocked with RPMI 1640 plus 10% FBS (HyClone Corp., Logan, UT, USA). CD8^+^ T cells were purified from splenocytes of 12-week-old non-diabetic female NOD.*β*2*m^null^.HHD* mice treated with or without different peptides using the EasySep mouse CD8^+^ T cell isolation kit (StemCell Technologies, Vancouver, Canada). Purified CD8^+^ T cells (purity>90%, 2×10^5^ cells/well) were incubated with each peptide (50 µg/ml)-pulsed T2 cells (1×10^4^ cells/well) for 24 h at 37°C. After incubation, cells were removed and plates were processed according to the IFN-γ ELISPOT kit (MabTech) manufacturer’s instructions. Spots were counted using a spot reader system (Saizhi, Beijing, China).

### Proliferation Assay

Splenocytes (1×10^6^ cells/ml) freshly isolated from 12-week-old non-diabetic female NOD.*β*2*m^null^.HHD* mice were co-cultured with 10 μg/ml indicated peptides and 10 U/ml recombinant murine interleukin 2 (rmIL-2; Peprotech, Rocky Hill, NJ, USA), followed by twice weekly rmIL-2. After incubation at 37°C for 72 h, [^3^H] thymidine (1 μCi/well) was added for an additional 16 h of culture, and uptake of [^3^H] thymidine was determined using a liquid scintillation counter (Beckman Coulter, Brea, CA, USA).

### Real-Time RT-PCR

Pancreatic biopsy samples (n=6) from indicated peptide-treated NOD.*β*2*m^null^.HHD* mice at 12 weeks of age were lysed in Trizol reagent (Invitrogen, Carlsbad, CA, USA), and total RNA was extracted according to the manufacturer’s instruction. About 500 ng of total RNA was used for reverse transcription using a PrimeScript^®^ RT reagent Kit (TaKaRa, Shiga, Japan) in a volume of 10 μl, and products were detected using a SYBR^®^ Premix Ex Tap™ Kit (TaKaRa). Data were collected and quantitatively analyzed on an M×3000 P Real-Time PCR System (Stratagene, Austin, TX, USA). Values were normalized using β-actin as an endogenous internal standard. The sequences used were as follows: mouse IL-6, Sense 5’-TAGTCCTTCCTACCCCAATTTCC-3’, Anti-sense 5’-TTGGTCCTTAGCCACTCCTTC-3’; mouse IL-1β, Sense 5’-GCAACTGTTCCTGAACTCAACT-3’, Anti-sense 5’-ATCTTTTGGGGTCCGTCAACT-3’; mouse TNF-α, Sense 5’-CACGCTCTTCTGTCTACTGAAC-3’, Anti-sense 5’-ATCTGAGTGTGAGGGTCTGG-3’; mouse IFN-γ, Sense 5’-TCAAGTGGCATAGATGTGGAAG-3’, Anti-sense 5’-CGCTTATGTTGTTGCTGATGG-3’; mouse IL-17, Sense 5’- ATCTGTGTCTCTGATGCTGTTG-3’, Anti-sense 5’- AACGGTTGAGGTAGTCTGAGG-3’; mouse β-actin, Sense 5’-GAGACCTTCAACACCCCAGC-3’, Anti-sense 5’-ATGTCACGCACGATTTCCC-3’.

### Isolation of Pancreas-Infiltration Cells

Mice were euthanized and systemically perfused by injection of physiological saline into the left heart ventricle. After removal of all visible pancreatic lymph nodes, the pancreases were cut into tiny pieces and then digested in HBSS containing 1 mg/ml collagenase IV and 1.25 μg/ml DNase (Sigma), by shaking (200 rpm) at 37°C for 15 min. Single-cell suspensions were collected after diluting the enzyme with ice-cold HBSS containing 2% FCS. Aggregates were further digested with 0.5 mg/ml collagenase IV and 1 μg/ml DNase for 10 min and 0.25 mg/ml collagenase IV and 1 μg/ml DNase for 6 min. Single-cell suspensions were washed three times, and pancreas-infiltration cells were isolated by percoll density-gradient centrifugation according to the manufacturer’s instructions, and then resuspended in medium.

### Flow Cytometry

Single-cell suspensions were made from spleens and pancreas-infiltrating immune cells of 12-week-old non-diabetic female NOD.*β*2*m^null^.HHD* mice immunized with different peptides and resuspended in RPMI 1640 medium. Fluorochrome-conjugated antibodies specific for surface markers used in this study were anti-CD3-FITC (145-2C11), anti-CD4-PE (GK1.5), and anti-CD8-PerCP-Cy5.5 (53-6.7) (eBiosciences, San Diego, CA, USA). Events were collected on the BD Acurri C6 flow cytometer and analyzed with FlowJo software.

### Statistical Analyses

Paired t-test was used to compare autoreactive CD8^+^ T cell responses to control peptide or mInsA_2-10_ of an individual within a certain treatment group. A non-parametric ANOVA (Kruskal-Wallis test) followed by a Dunn’s test was performed to analyze differences among the groups, and a log-rank test was used to assess the cumulative incidence of diabetes. Other statistical analyses were conducted by the two-tailed Student’s t-test. *P*<0.05 were considered statistically significant.

## Results

### The Treatment of Native mInsA_2-10_ Epitope Fails to Prevent the Development of T1D in NOD.*β2m^null^.HDD* Mice

**Figure 1:**
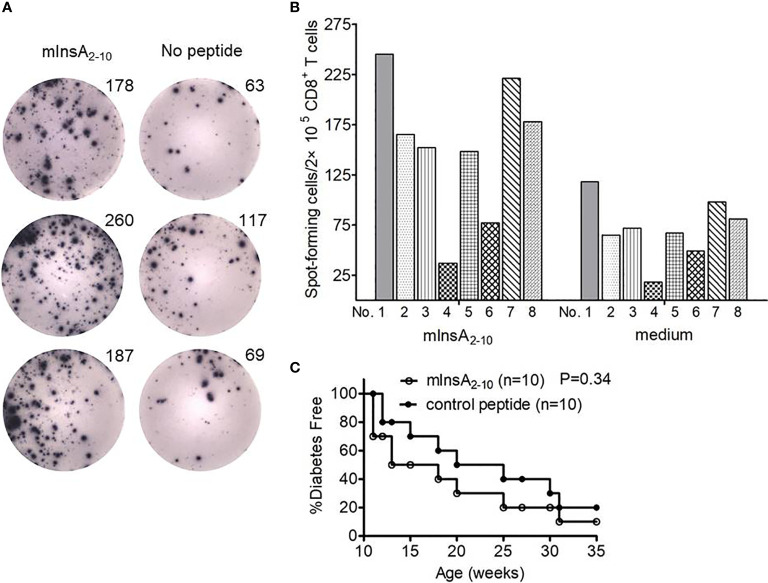
The mInsA_2-10_ epitope treatment could not prevent the development of T1D in NOD.*β*2*m^null^.HHD* mice. **(A)** Representative IFN-γ ELISPOT assay demonstrated splenic CD8^+^ T cell responses to T2 cell-loaded mInsA_2-10_ (10 μg/ml), or no peptide in 12-week-old non-diabetic female NOD.*β2m^null^.HHD* mice. **(B)** The average number of peptide-specific IFN-γ-positive spots per 2×10^5^ splenic CD8^+^ T cells in triplicate cultures was calculated for each indicated peptides (10 μg/ml) or no peptide from eight separate experiments. SDs are not shown to avoid excessive visual clutter. **(C)** Percentage of female NOD.*β2m^null^.HHD* mice developing diabetes after 100 μg mInsA_2-10_ i.p. weekly injections from 4 to 9 weeks of age (white circles, n = 10) *vs.* mice that were treated with OVA_257-264_ (black circles, n = 10).

We firstly verified the immunodominance of HLA-A*0201-restricted native mInsA_2-10_ peptide in 12-week-old non-diabetic female NOD.*β*2*m^null^.HHD* mice. As expected, IFN-γ ELISPOT analysis revealed that potent CD8^+^ T-cell responses against mInsA_2-10_ were indeed present in 12-week-old non-diabetic female NOD.*β*2*m^null^.HHD* mice (**Figures 1A, B**). So we questioned whether systemic administration of the native mInsA_2-10_ peptide could protect from T1D in NOD.*β*2*m^null^.HHD* mice. However, mInsA_2-10_ peptide showed no protective activity **(** **Figure 1C**), and this encouraged us to explore the APLs of mInsA_2-10_ peptide with antidiabetic activity.

### *In Silico* Rational Design of APLs of mInsA_2-10_ Epitope With a Single D-amino Acid Substitution

**Figure 2:**
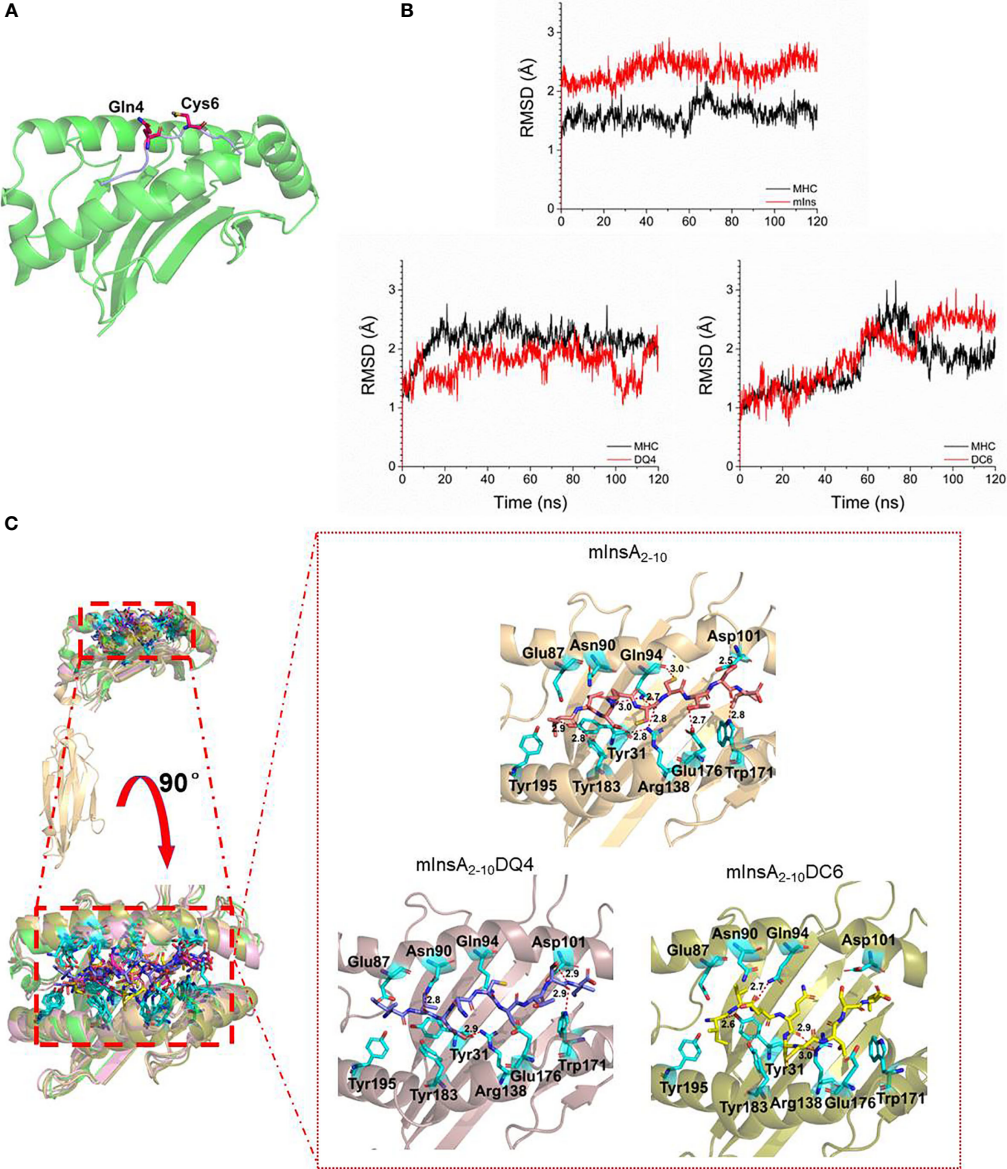
Design of a potential antagonist peptide for mInsA_2-10_. **(A)** The modeled structure of the HLA-A*0201/mInsA_2-10_ complex showed that residues Gln 4 and Cys 6 bulged out of the binding groove, which were the potential TCR contact residues. **(B)** The RMSD of simulation of complex HLA-A*0201/epitope, the stable conformation for these three complexes were achieved through 120 ns MD simulation, and the RMSD fluctuated near 2Å. **(C)** The binding mode between HLA-A*0201 and epitopes, these epitopes bound to HLA-A*0201 through stronger hydrogen bonds, but mInsA_2-10_ had the most hydrogen bonds.

To define the potential TCR contact residues in mInsA_2-10_, we constructed the HLA-A*0201/mInsA_2-10_(IVDQCCTSI) complex based on the crystal structure (PDB ID: 3MRE) by *in silico* replacing of epitope. **Figure 2A** showed amino acid residues at positions 4 and 6 were bulged out of the binding groove and potentially important for the interaction with TCR. Therefore, we generated two APL candidates *via in silico*-assisted replacing L-amino acid of mInsA_2-10_ at positions 4 and 6 by the corresponding D-isomer, respectively. Then, 120 ns MD simulation for the complex of HLA-A*0201/each peptide was performed, and the average structures (200 conformations) for the last 20 ns MD simulation were used to analyze their binding mode. The distance (C_α_) between amino acids at positions 1 and 9, RMSD, binding energy, and the number of hydrogen bonds between each peptide and HLA-A*0201 (**Table 1**), as well as the solvent accessible surface area (**Table 2**) and the conformation comparison between APL candidates and mInsA_2-10_, were analyzed. The RMSD plot showed that both HLA-A*0201 and mInsA_2-10_ remained stable after 80 ns MD simulation, with the fluctuation around 1.8 Å. While HLA-A*0201 molecule complexed with APL DQ4 and DC6 reached stable after 90 ns (**Figures 2B** **, C**) shows mInsA_2-10_ may stably bind to HLA-A*0201 through strong hydrophilic interaction. Notably, the predicted binding free energy of mInsA_2-10_DQ4 to HLA-A*0201 was very similar to that of mInsA_2-10_ to HLA-A*0201. Whereas, the predicted binding free energy between DC6 and HLA-A*0201 was increased (**Table 1**), indicating that the binding strength between DC6 and HLA-A* 0201 might be weakened. Thus, these results suggested that the substitution of D amino acid at position 4 rather than position 6 might have minor effect on the binding ability of peptide to HLA-A*0201 molecule.

**Table 2:** The solvent accessible surface area (SASA) of residues of epitopes.

Epitope	P1	P2	P3	P4	P5	P6	P7	P8	P9
mInsA_2-10_	−51.63	−1.04	2.33	122.81	40.35	49.28	55.88	63.21	29.50
mInsA_2-10_DQ4	−23.04	11.34	16.96	135.49	64.11	53.20	42.59	62.30	59.90
mInsA_2-10_DC6	−48.99	2.16	87.05	56.82	−1.53	61.45	6.53	82.70	80.73

P_i_ is position i.

### *In Vitro* Analysis of the T-Cell Stimulating Potency of the Selected APL Candidates of mInsA_2-10_

**Figure 3:**
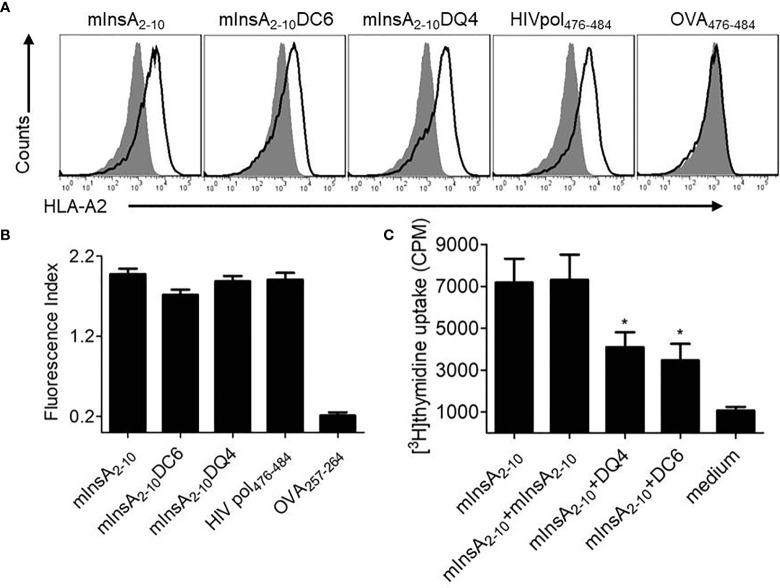
Selection of a promising antagonist peptide for mInsA_2-10_. **(A)** T2 cells were incubated with or without the indicated peptides (50 μg/ml), and the stabilization of surface HLA-A2 molecules was detected by flow cytometry. The HLA-A*0201-binding peptide HIV Pol_476-484_ was used as a positive control. The H-2K^b^-binding peptide OVA_257-264_ was used as a negative control. Filled histograms, no peptide; open histograms, plus peptide. **(B)** The binding affinity was presented as the fluorescent index (FI) that was calculated as follows: FI = (mean fluorescence intensity with the given peptide − mean fluorescence intensity without peptide)/(mean fluorescence intensity without peptide). Bars represent the mean ± SEM of three independent experiments. **(C)** Splenocytes were treated with 10 μg/ml mInsA_2-10_ plus 10 μg/ml indicated peptides for 72 h in 96-well plates (2×10^5^ per well) at 37°C. The cell proliferation was measured by [^3^H] thymidine incorporation. Bars represent the mean ± SEM of seven independent experiments. *P < 0.05.

The binding affinity of the native peptide mInsA_2-10_ and the two APLs for HLA-A*0201 molecule was evaluated *in vitro* using a T2-cell-peptide binding test. Both mInsA_2-10_ and mInsA_2-10_ DQ4 showed a strong HLA-A*0201-binding affinity similar to the positive control HIVpol_476-484_. Whereas mInsA_2-10_DC6 showed a relatively weaker binding affinity to HLA-A*0201 (**Figures 3A, B**). These results confirmed that the substitution of D-amino acid at position 4 rather than position 6 did not change the binding ability of APL to HLA-A*0201. To assess whether mInsA_2-10_DQ4 and DC6 displayed any stimulating or inhibiting activity toward mInsA_2-10_-reactive T cell population, the proliferation of splenocytes from 12-week-old non-diabetic female NOD.*β2m^null^.HHD* mice was tested upon the stimulation with mInsA_2-10_ alone or plus each APL. When compared to the stimulation with mInsA_2-10_ alone, the proliferation of splenocytes decreased significantly upon stimulation with mInsA_2-10_ plus either mInsA_2-10_ DQ4 or DC6 (**Figure 3C**). Thus, these preliminary data indicated that both mInsA_2-10_DQ4 and DC6 displayed inhibitory effects on mInsA_2-10_-stimulated lymphocyte proliferation *in vitro*.

### The Treatment of mInsA_2-10_DQ4 Reduces Insulitis and Prevents the Development of T1D in NOD.*β2m^null^.HHD* Mice

**Figure 4:**
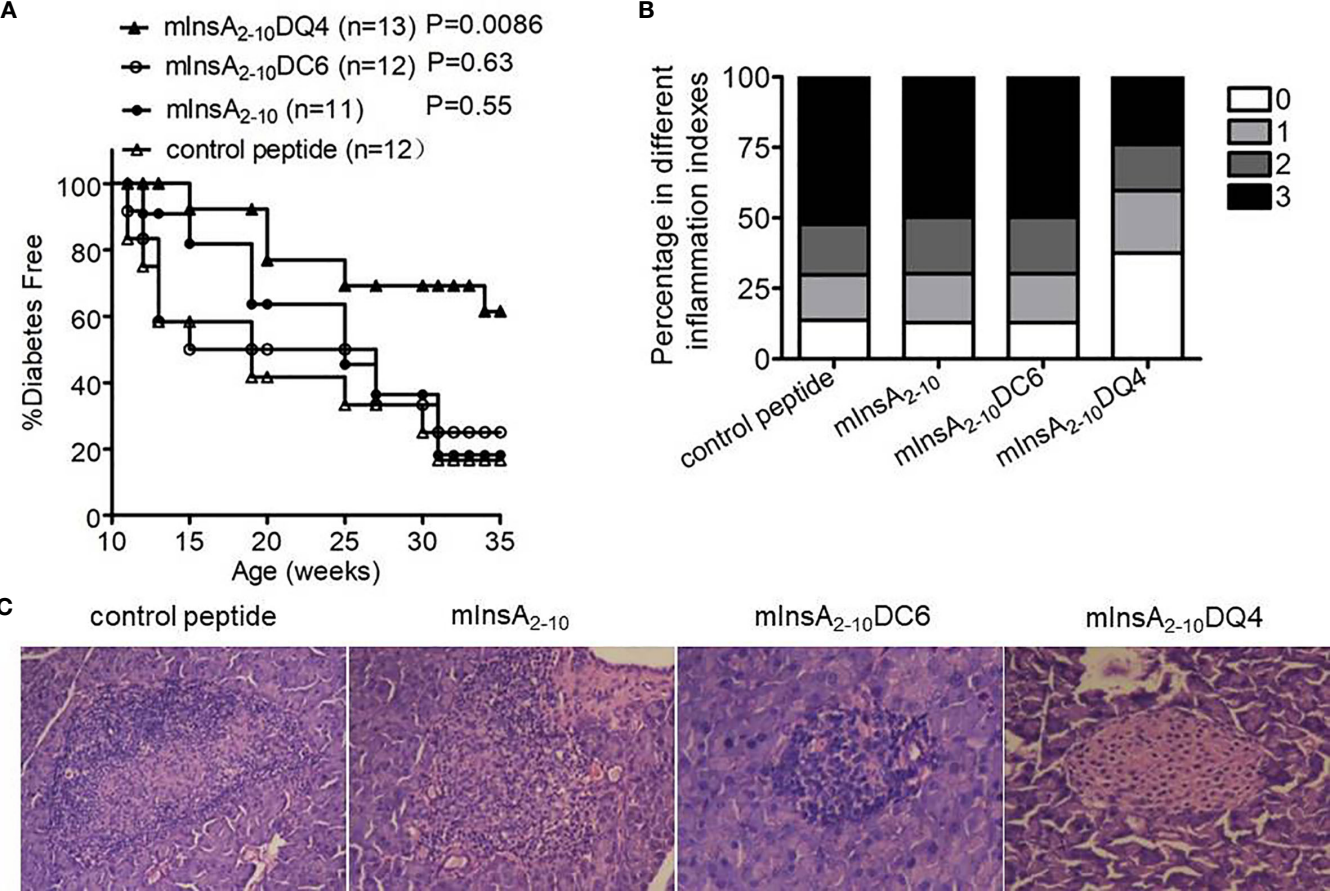
The treatment with mInsA_2-10_DQ4 prevented diabetes development in NOD.*β*2*m^null^.HHD* mice. NOD.*β*2*m^null^.HHD* female mice were injected intraperitoneally (once weekly) at 4–9 weeks of age with 100 μg of peptide mInsA_2-10_, mInsA_2-10_DQ4, mInsA_2-10_DC6, or OVA_257-264_. **(A)** Mice were monitored for diabetes development. **(B)** Histopathological evaluation of pancreatic sections from indicated peptide-treated NOD.*β*2*m^null^.HHD* mice at 12 weeks of age. **(C)** Representative hematoxylin and eosin–stained paraffin-embedded pancreas sections (200 × magnification) are shown.

To investigate whether the two APLs had antidiabetic activity *in vivo*, female NOD.*β2m^null^.HHD* mice were injected intraperitoneally with soluble peptide mInsA_2-10_, mInsA_2-10_DC6, mInsA_2-10_DQ4, or control peptide (OVA_257-264_) in PBS, respectively, starting at 4 weeks of age. Consistently, native mInsA_2-10_ peptide treatment had no protective effect on T1D. Interestingly, administration of mInsA_2-10_DQ4, but not DC6, significantly suppressed the development of T1D compared with the control group (*p*=0.0086) (**Figure 4A**). Histopathological analysis of mInsA_2-10_DQ4-treated female NOD.*β2m^null^.HHD* mice at 12 weeks showed less insulitis, compared to age-matched non-diabetic NOD.*β2m^null^.HHD* mice of mInsA_2-10_, mInsA_2-10_DC6, or control peptide treatment (**Figures 4B, C**). These results indicated that the treatment of mInsA_2-10_DQ4 reduces insulitis and prevents the development of T1D in NOD.*β2m^null^.HHD* mice.

### The Treatment of mInsA_2-10_DQ4 Results in Loss of mInsA_2-10_ Autoreactive CD8^+^ T Cell Responses in NOD.*β2m^null^.HHD* Mice

**Figure 5:**
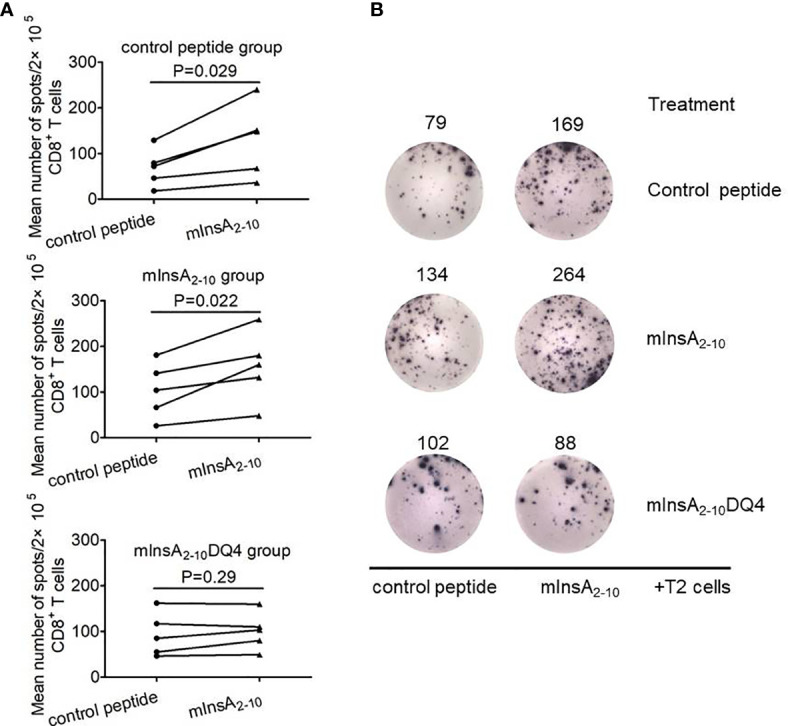
mInsA_2-10_DQ4 treatment resulted in loss of mInsA_2-10_ autoreactive CD8^+^ T cell responses. **(A)** Statistical analysis of the average number of control peptide or mInsA_2-10_-specific IFN-γ-positive spots per 2×10^5^ splenic CD8^+^ T cells in triplicate cultures with a treatment group (n = 5) are shown. Significance was determined by a paired *t*-test. **(B)** Representative IFN-γ ELISPOT assay demonstrated splenic CD8^+^ T cell responses to T2 cell loaded with mInsA_2-10_ in each peptide-treated group of non-diabetic NOD.*β*2*m^null^.HHD* mice at 12 weeks of age.

To further determine whether mInsA_2-10_DQ4 treatment could induce mInsA_2-10_-specific CD8^+^ T cell tolerance *in vivo*, we analyzed mInsA_2-10_-pulsed T2 cells stimulated IFN-γ spots forming by purified splenic CD8^+^ T cells from NOD.*β2m^null^.HHD* mice treated with mInsA_2-10_DQ4, mInsA_2-10_, or control peptide. As shown in **Figures 5A** ,**B**, when compared with control peptide-loaded T2 cells, the stimulation of T2 cells pulsed with mInsA_2-10_ significantly increased IFN-γ spot formation of splenic CD8^+^ T cells in both control peptide and mInsA_2-10_-treated NOD.*β2m^null^.HHD* mice. In contrast, T2 cells pulsed with mInsA_2-10_ failed to increase IFN-γ spot formation of splenic CD8^+^ T cells in mInsA_2-10_DQ4-treated NOD.*β2m^null^.HHD* mice. These results together indicated that treatment with mInsA_2-10_DQ4 blinded peripheral mInsA_2-10_-autoreactive CD8^+^ T cell responses in NOD.*β2m^null^.HHD* mice.

### The Treatment of mInsA_2-10_DQ4 Reduces the Infiltration of CD4^+^ T and CD8^+^ T Cells and Inflammatory Responses in the Pancreas of NOD.*β2m^null^.HHD* Mice

**Figure 6:**
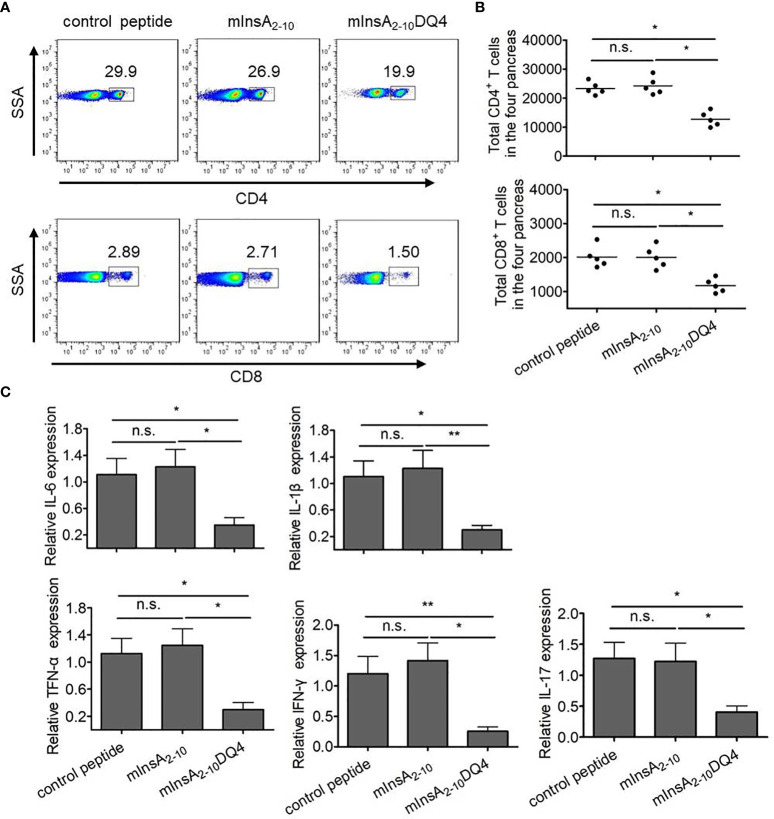
Treatment with mInsA_2-10_DQ4 notably reduced frequencies and absolute numbers of pancreas-infiltrating CD4^+^ T and CD8^+^ T cells in NOD.*β*2*m^null^.HHD* mice. **(A)** Representative fluorescence-activated cell sorting (FACS) histogram plots for pancreas-infiltrating CD4^+^ T and CD8^+^ T cells from four mice. **(B)** The results for absolute numbers of these pancreas-infiltrating CD4^+^ T and CD8^+^ T cells are expressed as the mean ± SD. Each symbol in **(B)** represents a sample of pooled pancreas-infiltrating CD4^+^ T and CD8^+^ T cells from four mice. **(C)** Total RNA was extracted from the pancreas in each peptide-treated group of non-diabetic mice (n = 6). The mRNA expression levels of these cytokines were quantified by real-time RT-PCR. The data are presented as fold-change compared to the mRNA levels expressed in pancreas from control peptide-treated mice. *P < 0.05 and **P < 0.01. n.s. indicates no significance.

Since insulin has been considered as an only essential autoantigen to initiate spontaneous T1D and the elimination of insulin-reactive T cells can block epitopes expansion and subsequent destruction of β cells by other autoreactive T cells in NOD mice, we questioned whether the treatment of mInsA_2-10_DQ4 prevented the infiltration of CD4^+^ and CD8^+^ T cells and inflammatory responses in the pancreas of *NOD.β2m^null^.HHD* mice. Strikingly, both the frequency and absolute number of pancreas-infiltrating CD4^+^ T and CD8^+^ T cells were markedly reduced in mInsA_2-10_DQ4-treated NOD.*β2m^null^.HHD* mice (CD4^+^ T cells, 18.5 ± 1.7%, 12,757.4 ± 2,556.6; CD8^+^ T cells, 1.6 ± 0.3%, 1,172.2 ± 206.1) compared with those detected in mInsA_2-10_-treated (CD4^+^ T cells, 29.3 ± 4.0%, 24,215.2 ± 2,994.9; CD8^+^ T cells, 3.3 ± 0.4%, 2,005.8 ± 326.6) or control peptide-treated (CD4^+^ T cells, 29.2 ± 5.7%, 23,309.2 ± 2,171.7; CD8^+^ T cells, 3.2 ± 0.5%, 2,015.2 ± 314.6) NOD.*β2m^null^.HHD* mice (**Figures 6A, B**). As expected, the mRNA levels of the proinflammatory cytokines IL-6, IL-1β, TNF-α, IFN-γ, and IL-17 significantly decreased in the pancreas of mInsA_2-10_DQ4-treated NOD.*β2m^null^.HHD* mice, when compared with NOD.*β2m^null^.HHD* mice that received mInsA_2-10_ or control peptide injection (**Figure 6C**). Thus, these data suggested that the treatment of mInsA_2-10_DQ4 reduces the aggregation of autoreactive CD4^+^ T and CD8^+^ T cells and inflammatory responses in the pancreas of NOD.*β2m^null^.HHD* mice.

## Discussion

T1D in both NOD mice and humans is an organ-specific autoimmune disease resulting from selective damage of pancreatic β cells by autoreactive T lymphocytes. Autoreactive CD8^+^ T cells, which play an indispensable key role in initiation and progression of T1D, are recommended as ideal targets for the prevention of T1D. In this study, we generated an APL, mInsA_2-10_DQ4, derived from D-amino acid substitution at a potential TCR contact site of an HLA-A*0201-restricted immunodominant insulin epitope. Treatment with mInsA_2-10_DQ4 significantly suppressed the development of T1D in NOD.*β2m^null^.HHD* mice *via* inducing mInsA_2-10_ autoreactive CD8^+^ T cell tolerance and preventing the subsequent infiltration of CD4^+^ T and CD8^+^ T cells as well as inflammatory responses in the pancreas in NOD.*β2m^null^.HHD* mice.

It has been reported that the modification of peptides with D-amino acids instead of natural L-amino acids can not only preserve the feasible recognition properties but also significantly improve the stability and half-life of peptides (23, 24). Most of the available literatures reported that the replacement of all L-amino acid residue in T cell epitope by their D-enantiomers results in normal or reversed (retro) amide linkage (25, 26). A recent study reported that a retro-inverso-D-amino acid-based insulin B-chain (9–23) peptide blocked the presentation of native InsB_9-23_ by HLA-DQ8 to autoreactive T-cells, suggesting that D-amino acid peptides may be an innovative treatment for T1D (27). Whereas, relatively few studies have been reported on a single amino acid substitution by the respective D-enantiomer in T cell epitope. For example, the individual substitutions by the corresponding D-amino acid at most positions within an I-E^d^-restricted 13-mer snake toxin epitope greatly diminished its T-cell stimulating activity (17). A modified H-2D^d^-restricted epitope of HIV-1 IIIB envelope glycoprotein P18-I10 (RGPGRAFVTI) with a single amino acid substitution by the respective D-enantiomer at positions 324F, 325V, 326T, or 327I markedly reduced the cytotoxic activity of P18-I10-specific murine CD8^+^ T lymphocytes (18).

Subtle changes at the TCR contact residues can dramatically alter the downstream signaling events, leading to T-cell anergy, apoptosis, or high activation (28). Since amino acid residues at positions 4 and 6 of mInsA_2-10_ were predicted to be in close contact with the TCR, we generated two APL candidates *via in silico*-assisted single D-amino acid substitution at the two positions, respectively. Our *in silico* analysis predicted that single D-amino acid substitution at position 4 rather than position 6 might have minor influence on the binding affinity of epitope to HLA-A*0201. These simulation results were supported by the peptide binding assays, which showed that DQ4 had a similar HLA-A*0201-binding affinity with that of native peptide, whereas DC6 displayed a relatively weaker binding ability to HLA-A*0201. Although both two APLs showed antagonistic effects against natural peptide-stimulated T cell proliferation *in vitro*, only mInsA_2-10_DQ4, but not DC6, exhibited *in vivo* antidiabetic effects in NOD.*β2m^null^.HHD* mice. A plausible explanation is that APL DC6 has a reduced binding ability to HLA-A*0201 relative to its parent peptide, and it is difficult for DC6 to competitively bind to HLA-A*0201 *in vivo* and therefore cannot be an effective antagonist. Whereas, mInsA_2-10_DQ4 not only has a similar HLA-A*0201-binding affinity with mInsA_2-10_ but also has a theoretically stronger stability *in vivo* than mInsA_2-10_. Therefore, DQ4 could effectively antagonizes the natural peptide *in vivo.* The metabolic dynamics and stability of DQ4 need further study.

We did not further study the mechanisms by which DQ4 induced mInsA_2-10_-autoreactive T-cell tolerance in humanized NOD mice. APLs with amino acid substitutions at TCR contact positions are suggested as useful tools to modulate T cell responses induced by the native peptide. For example, APLs of insulin B_15-23_ (LYLVCGERG) G6H and R8L, which were generated by one natural amino acid substitution at position 6 and position 8 (TCR contact sites), respectively, showed the antagonist activity of the highly pathogenic insulin B_15-23_-reactive CD8^+^ T cell clone G9C8 in cytotoxicity and IFN-γ production assays (29). Another study indicated that a superagonist APL with an amino acid substitution at position 6 TCR contact site proved more effective than the native peptide in blocking autoimmune diabetes by a decreased accumulation of pathogenic CD8^+^ T cells in the pancreas (30). We hypothesized that DQ4 with an increased stability and modified TCR contact site could provide an altered antigen stimulation signal to the native mInsA_2-10_-autoreactive T cells, leading to induce their anergy or apoptosis, or change their functional state *in vivo*. However, the specific molecular and cellular mechanisms need to be further studied.

At least two studies have reported the presence of specific T cell responses to human proinsulin (PPI) _90-99_ or InsA_1-10_ (GIVEQCCTSI) in T1D patients (31, 32). Thus, human InsA_2-10_ is likely to be an immunogenic target for diabetic patients. The sequence of mInsA_2-10_ (IVDQCCTSI) is highly consistent with that of hInsA_2-10_ (IVEQCCTSI) with only one amino acid difference at position 3, suggesting the possibility of cross-reactivity between the two peptides in T1D patients, but further studies are needed. We also did not detect whether mInsA_2-10_ DQ4 can antagonize T cell responses towards hInsA_2-10_ in PBMC of T1D patients. Therefore, further studies are needed to determine whether DQ4 has potential clinical application value.

As we know, prediction of T1D onset is possible, and prevention is now a goal in T1D. T1D vaccines based on islet autoantigens are considered to be one of such strategies to prevent or delay the occurrence of T1D by modulating autoimmune responses towards pancreatic islet antigens and prevent further destruction of pancreatic β-cells in T1D-susceptible individuals or preclinical individuals (33). Interestingly, our findings confirmed again that insulin is the key autoantigen for T1D initiation, since the administration of DQ4 not only eliminated peripheral mInsA_2-10_-autoreactive T cells response but also reduced the subsequent infiltration of CD4^+^ T and CD8^+^ T cells as well as inflammatory responses in the pancreas in NOD.*β2m^null^.HHD* mice. Consistent with this, administration of chemically fixed splenic antigen-presenting cells coupled with intact insulin or the dominant insulin epitopes, but not epitopes of other autoantigens, protected 4–6-week-old NOD mice from development of T1D, which also indicated insulin is the key initiating autoantigen (34). Therefore, induction of insulin-autoreactive T cell immune tolerance in prediabetic mice may reduce the autoimmune insulitis and damage of islets, and prevent the subsequent epitope spreading and the activation and recruitment of other autoreactive T cells. In this sense, T1D vaccine targeting insulin, such as APLs of insulin epitopes, has potential value in the prevention of T1D when applied to the high-risk individuals whose islet autoimmunity has not yet occurred.

In conclusion, this present study describes that an APL designed by *in silico*-assisted single D-amino acid substitution at the potential TCR contact site displayed a protective effect against T1D in humanized NOD mice. This study provides a new strategy for the development of APL vaccines for T1D prevention.

## Data Availability Statement

The original contributions presented in the study are included in the article/supplementary material. Further inquiries can be directed to the corresponding author.

## Ethics Statement

The animal study was reviewed and approved by the Animal Ethics Committee of the Army Medical University (Third Military Medical University).

## Author Contributions

MZ and YW performed main experiments and data analysis and drafted the manuscript. XL, GM, XC, and LNW performed experiments and/or analyzed data. ZL revised the manuscript critically for important intellectual content. LW carried out the project design, guided the research process, supervised and managed it, completed the manuscript, and provided research funds. All authors contributed to the article and approved the submitted version.

## Funding

This work was supported by the National Natural Science Foundation of China (Nos. 31771002, 81871301, and 82071825) and the National Key Project for Research & Development of China (No. 2016YFA0502204).

## Conflict of Interest

The authors declare that the research was conducted in the absence of any commercial or financial relationships that could be construed as a potential conflict of interest.

## Publisher’s Note

All claims expressed in this article are solely those of the authors and do not necessarily represent those of their affiliated organizations, or those of the publisher, the editors and the reviewers. Any product that may be evaluated in this article, or claim that may be made by its manufacturer, is not guaranteed or endorsed by the publisher.
